# Accumulation of evidence during decision making in OCD patients

**DOI:** 10.3389/fpsyt.2022.980905

**Published:** 2022-09-23

**Authors:** Yilin Chen, Ying Liu, Zhen Wang, Tianming Yang, Qing Fan

**Affiliations:** ^1^Key Laboratory of Primate Neurobiology, CAS Center for Excellence in Brain Science and Intelligence Technology, Institute of Neuroscience, Chinese Academy of Sciences, Shanghai, China; ^2^University of Chinese Academy of Sciences, Beijing, China; ^3^Shanghai Mental Health Center, Shanghai Jiao Tong University School of Medicine, Shanghai, China; ^4^Shanghai Center for Brain Science and Brain-Inspired Intelligence Technology, Shanghai, China; ^5^Shanghai Key Laboratory of Psychotic Disorders, Shanghai, China

**Keywords:** obsessive-compulsive disorder (OCD), decision-making, evidence accumulation, drift diffusion model, probabilistic reasoning

## Abstract

Decision-making often entails the accumulation of evidence. Previous studies suggested that people with obsessive-compulsive disorder (OCD) process decision-making differently from healthy controls. Both their compulsive behavior and obsessive thoughts may influence the evidence accumulation process, yet the previous studies disagreed on the reason. To address this question, we employed a probabilistic reasoning task in which subjects made two alternative forced choices by viewing a series of visual stimuli. These stimuli carried probabilistic information toward the choices. While the OCD patients achieved similar accuracy to the control, they took longer time and accumulated more evidence, especially in difficult trials in which the evidence strength was low. We further modeled the subjects' decision making as a leaky drifting diffusion process toward two collapsing bounds. The control group showed a higher drifting rate than the OCD group, indicating that the OCD group was less sensitive to evidence. Together, these results demonstrated that the OCD patients were less efficient than the control at transforming sensory information into evidence. However, their evidence accumulation was comparable to the healthy control, and they compensated for their decision-making accuracy with longer reaction times.

## Introduction

Obsessive-compulsive disorder (OCD) is a common psychiatric disorder in which a patient experiences either uncontrollable intrusive thoughts, repetitive behaviors, or both (1). A broad spectrum of behavior anomalies have been reported in OCD patients, including but not limited to deficits in working memory and long-term memory (2–6), intolerance of uncertainty (7–18), and abnormal motor inhibition (19). Understanding the neural substrate of these behavior anomalies is essential, as neural modulation treatment targeting specific brain regions has been growing more common. Appropriate behavior paradigms, especially those whose neural mechanisms have been carefully studied in humans and animals, may help reveal relevant neural circuitry in OCD patients' altered behavior.

In particular, numerous studies have been done to advance the understanding of the decision-making process in OCD patients, yet the results were mixed. Some studies showed that OCD patients had lower decision accuracy than the controls (20, 21) while others showed the opposite (22, 23). Besides the choice accuracy, it has been reported that OCD patients were slow in committing to a decision relative to the controls and accumulated more evidence in the process (20–24). However, this oversampling behavior among OCD patients was not replicated in several other studies (25, 26). Some even observed impulsive behavior in OCD patients, whose decisions were often hasty, risky, and based on weak evidence (27).

The inconsistency among these studies reflects the complex nature of decision-making. The decision-making process could be segmented into several stages, including sensory-to-evidence transformation, evidence accumulation, decision threshold crossing, and motor planning and execution. Different cognition processes and their corresponding neural circuitries are engaged during each stage. It has been shown that the orbitofrontal cortex (Sirunyan et al.) computes and transforms visual sensory inputs into evidence, and a frontoparietal network, including the dorsolateral prefrontal cortex and the posterior parietal cortex, is involved in integrating evidence in decision making (28–31). For OCD, many studies have also pointed to a prominent role of the OFC in OCD symptoms. It has been suggested that an unbalanced activation of the orbitofrontal-striatal circuit underlies OCD (32). Deep brain stimulation (DBS) studies in OCD patients show that targeting fibers from the OFC to the striatum leads to better treatment effects (33). Furthermore, optogenetic stimulations of the Sapap3 mutant mice's lateral OFC (lOFC) neurons successfully inhibited their OCD-like repetitive self-grooming behavior (34). These results lead to our speculation that the sensory-evidence transformation during decision-making in OCD patients is compromised.

To test this hypothesis, we used a probabilistic reasoning task adapted from earlier animal studies to investigate the evidence accumulation process in OCD patients' decision-making (35, 36). This task paradigm is advantageous over typical perceptual decision-making paradigms, such as the random dots motion task (24, 37–39), which are based on the discrimination of noisy stimuli and less ideal for isolating the cognitive component of decision making. In the task, the subjects needed to accumulate probabilistic evidence based on a sequence of visual stimuli to perform a two-alternative forced-choice task. The stimuli were easily discriminable arrow shapes of different directions and contrasts. Each stimulus provided a piece of evidence toward the choices, and the subjects needed to accumulate the evidence for making appropriate decisions. We studied the OCD patients' performance and how stimuli and their associated evidence contributed to decisions. We found that the OCD patients were less sensitive to individual pieces of evidence than the healthy controls, yet they achieved a similar overall accuracy by collecting more evidence. As a result, their reaction times were significantly longer than healthy controls but only in the difficult trials. These results, combined with the previous findings of the prefrontal circuitry underlying evidence accumulation, hint an altered prefrontal – orbitofrontal in particular –functions underly decision-making in OCD patients.

## Method

### Participants

We collected behavior data from 28 healthy controls and 33 OCD patients at Shanghai Mental Health Center. The OCD patients' diagnosis, recruitment, and psychiatric tests (Yale-Brown Obsessive-Compulsive Scale (Y-BOCS) (40), Hamilton Anxiety Rating Scale (HAM-A) (41), and Hamilton Depression Rating Scale (HAM-D) (42) were performed by the psychiatric professionals from Shanghai Mental Health Center. The inclusion criteria of the OCD patients were: (a) between 18 and 54 years old, with no gender restrictions; (b) diagnosed as OCD based on DSM-IV criteria; (c) Y-BOCS ≥ 16 points; (d) not under any medication for at least 8 weeks; (e) education level no less than junior high school; (f) sufficient vision to accomplish the experiment, and (g) ability to provide the informed consent. The exclusion criteria were: (a) diagnosed with DSM-IV Axis I other than OCD; (b) severe physical disease, central nervous system disease, or substance abuse; c) pregnancy or nursing; (d) prominent suicidal ideation. The inclusion criteria for the control group were: (a) no obsessive and compulsive symptoms; (b) no psychotropic medication history; (c) no relatives within three generations of direct or collateral blood had any psychiatric disorder history; (d) sufficient vision to accomplish the experiment and (e) ability to provide the informed consent. The exclusion criteria of the control group were: (a) diagnosed with the DSM-IV Axis I psychiatric disorders; (b) diagnosed with the DSM-IV Axis II compulsive personality disorders; (c) pregnancy or nursing; (d) prominent suicidal ideation. The healthy control participants were recruited through open advertisement. All procedures followed the protocol approved by the Ethics Committee of Shanghai Mental Health Center (No. 2017-02) (Shanghai, China).

We calculated the accuracy of each subject and excluded those that were outside of the 95% confidence interval. With this exclusion criterium, 26 healthy controls and 32 OCD patients were included in the rest of the analyses. The accuracies of the two excluded healthy controls and the one OCD patient were 0.55, 0.53, and 0.47, respectively.

### Task paradigm

**Figure 1:**
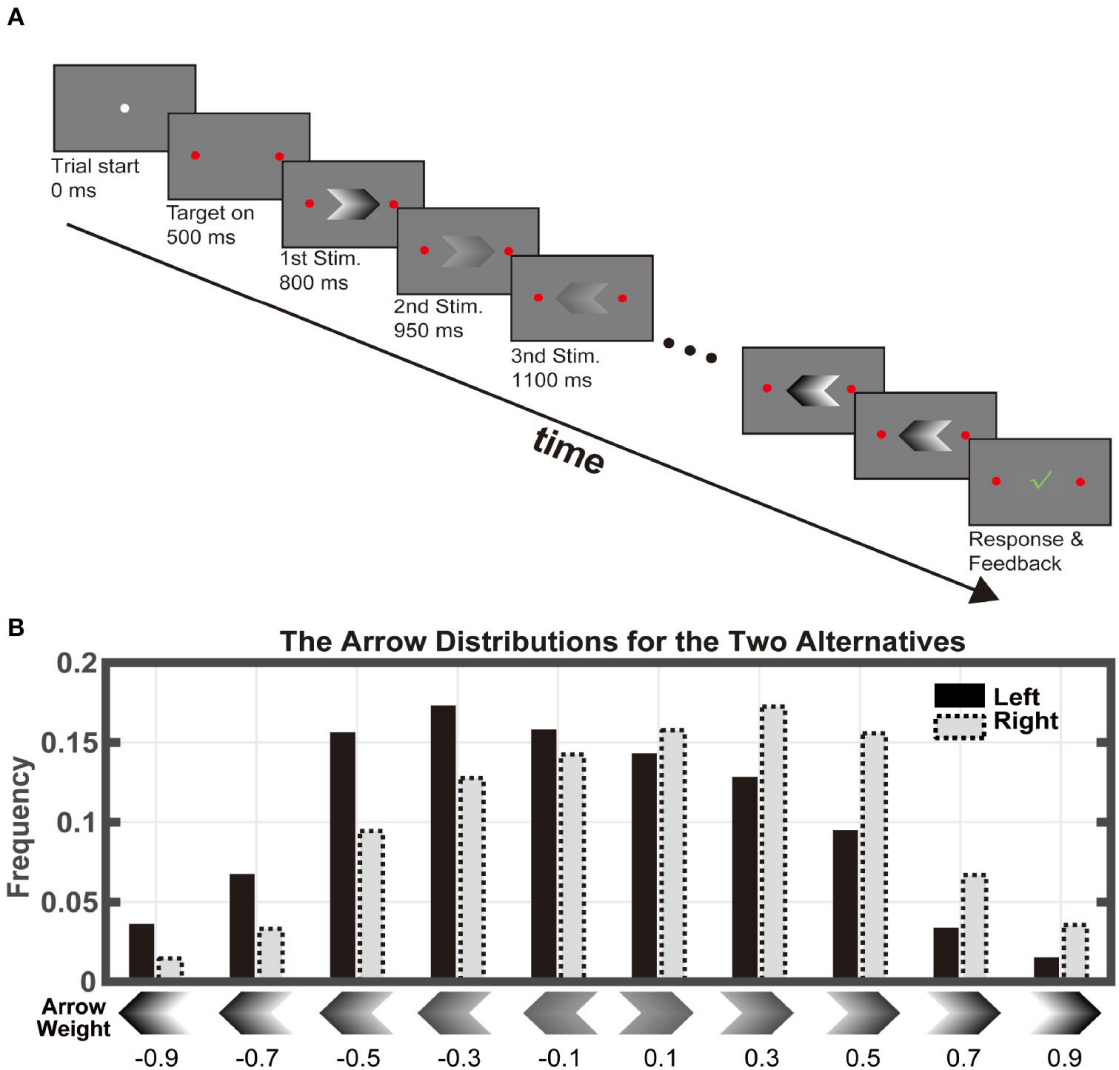
Task paradigm. **(A)** Subjects viewed a series of arrow stimuli at different contrasts. Each stimulus was displayed for 150ms without inter-stimulus-interval. The maximal stimulus length was 100. The subjects indicated their choice by pressing one of the two buttons any time during the stimulus viewing period. **(B)** There were 10 stimulus types: two directions at 5 contrast levels. The stimuli were drawn from the distribution associated with the correct answer. The numbers below indicate the weight assigned to each stimulus, which equals the log-ratio between the probabilities of that the stimulus appears when the right target is the correct answer and when the left target is correct.

The subjects made two-alternative forced choices by pressing a left or a right button with their fingers (Figure 1A). In each trial, a stream of visual stimuli appeared sequentially at the center of a computer screen. The stimuli were sampled from a stimulus set of 10 arrow shapes, which faced either left or right and had five different contrast levels (Figure 1B). The subjects were asked to judge, on average, which group of arrows had higher contrast by pressing the corresponding button.

Each arrow provides a piece of evidence toward the correct choice because its appearance probability depended on the choice. The probabilities of the arrows associated with the left choice were [0.044, 0.083, 0.15, 0.17, 0.22, 0.18, 0.084, 0.048, 0.017, and 0.006], and those with the right choice were [0.006, 0.017, 0.048, 0.084, 0.18, 0.22, 0.17, 0.15, 0.083, and 0.044]. Thereby, the evidence associated with the arrows can be quantified by the evidence weights, which are defined as the natural log-ratio between its appearance probability associated with the right choice and that associated with the left choice. They were −0.9, −0.7, −0.5, −0.3, −0.1, 0.1, 0.3, 0.5, 0.7, and 0.9 (Figure 1B). The five left arrows had negative weights; they were evidence favoring the left choice. The five right arrows had positive weights, which were in favor of the right choice. In addition, arrows with higher contrasts were associated with larger absolute evidence weights, indicating stronger evidence for their corresponding choices.

The trial sequence was as follows. A circle with a 25-pixel diameter appeared on the center of the screen indicating the trial start. After 0.5 s, two round targets with a diameter of 62.5 pixels appeared on the left and the right side of the central circle. After another 0.3 s delay, the central circle disappeared, and a serial of arrows was displayed at the center of the screen sequentially. Each arrow was presented for 150 ms with no inter-stimulus interval. The maximal length of the stimulus sequence was 100. The participants were allowed to make choices throughout the stimulus viewing period by pressing the left or the right key. Trials were aborted if a choice was not indicated before the maximum number of stimuli was run out. A green check mark would appear on the screen center for 0.5 s if the choice were correct. Otherwise, a red cross sign would appear instead. The subjects first took twenty practice trials and then completed a hundred test trials. The test session typically took <20 min. The subjects received a small amount of cash (RMB 80 yuan) for their participation in the experiment. The amount of payment was unrelated to their performance.

### Behavioral analyses

#### Non-decision stimulus number

The last few stimuli in each trial might not contribute to the decision due to the motor preparation and other downstream processes (35). To estimate the number of non-contributing stimuli, we modeled the subjects' choices by segmenting the arrow sequence into two:


(1) $$\begin{array}{l} choice=logit\left(\beta_{0}+\beta_{1}\overset{N-n_{nd}}{\underset{i=1}{\sum }}w_{i}+\beta_{2}\overset{n_{nd}}{\underset{i=1}{\sum }}w_{N-i+1}\right), \end{array}$$

where w_i_ is the weight of the *i*^*th*^ stimulus, *n*_nd_ is the number of non-contributing arrows, and *N* is the length of the stimulus sequence (*N* > *n*_nd)_. We began with *n*_nd_=1 and increased *n*_nd_ until β_2_ reached the significant level in the model. We defined the non-decision stimulus number using the largest *n*_nd_ without β_2_reaching significance and removed the last *n*_nd_ stimuli from the subject's trial sequences in the following analysis. This non-decision time was independently estimated for each participant. We used N^*^ to indicate the length of the adjusted stimulus sequence. Trials with length equal or <N^*^ were excluded in the rest of the analyses.

#### Psychometric curve and subjective value

The psychometric curve was a logistic regression fitting to the subjects' choice:


(2) $$\begin{array}{l} P\left(Choice=Right\right)=\frac{1}{1+e^{-Q}}, \end{array}$$

(3) $$\begin{array}{l} Q=\beta_{0}+\beta_{1}\overset{N^{*}}{\underset{i=1}{\sum }}w_{i}, \end{array}$$

where w_i_ represents the weight of the arrow in epoch *i*, β_0_is the bias term, and β_1_ is the estimate of how the total weight affected the choice. The estimate reflects the sensitivity of the subjects' choice to the accumulated evidence.

We performed another logistic regression to assess how each type of arrow affected the choice:


(4) $$\begin{array}{l} P\left(Choice=Right\right)=\frac{1}{1+e^{-Q}}, \end{array}$$

(5) $$\begin{array}{l} Q=\beta_{0}+\overset{10}{\underset{i=1}{\sum }}\beta_{i}N_{i}, \end{array}$$

where N_i_ represents how many times arrow type *i* appears in a trial. β_0_is the bias term, and $\beta_{1\tilde{\;}10}$are the estimates of how much leverage each type of arrow has on the subjects' choices and termed the subjective weights of evidence (36).

#### Trial difficulty

To measure the evidence strength of a trial, we regressed the accumulated evidence at each presentation of the arrows against its index with a linear model:


(6) $$\begin{array}{l} \overset{T}{\underset{i=1}{\sum }}w_{i}=\beta_{0}+\beta_{1}T, \end{array}$$

where $\overset{T}{\underset{i=1}{\sum }}w_{i}$ is the sum weight up to the *T-th* arrow (T = 1. N^*^). Thus, β_1_ is a measure of how fast the evidence is accumulated with which we defined the trial's evidence strength. The evidence strength is a signed number. The sign indicates the choice that the evidence favors, and the absolute value indicates the strength.

We divided all trials into 8 bins based on their evidence strength for analyses in **Figure 3**. We used smaller bins for evidence strengths smaller than 0.2 and larger bins for those higher than 0.2, because the trials with evidence strength smaller than 0.2 were over-represented in the data. Thereby, each bin has enough trials, while it does not cover too broad a range of evidence strength (Supplementary Table 1). Our conclusions did not rely on the particular choice of this binning method.

#### Drift diffusion model

We fitted each subject's choice and reaction time with the DDM using the pyddm toolbox (43). We included symmetric exponentially collapsing (rate τ) bounds (bound height B) and a leaky (leaky rate λ) integrator in the model. To speed up the fitting, we used a constant drifting rate in each trial, which was proportional to the evidence strength, with a sensitivity constant *C*. A non-decision time parameter (t_nd_) was also included. Additionally, we included a fixed lapse term *L*, which is the proportion of trials with choices generated from an evidence-independent Poisson process with the rate = 1/sec.

#### History effects

The influence of the trial history on the current choice (left/right) was studied with logistic regression:


(7) $$\begin{array}{l} choice=logit\left(\beta_{0}+\beta_{1}\overset{N^{*}}{\underset{i=1}{\sum }}w_{i}+\beta_{2}C_{I}+\beta_{3}R_{I}+\beta_{4}C_{I}\times R_{I}\right), \end{array}$$

where $\overset{N^{*}}{\underset{i=1}{\sum }}w_{i}$ is the accumulated evidence of the current trial. C_I_ is the last trial's choice (left/right), R_I_ is the outcome of the last trial (correct/wrong), and the last term is the interaction between the two.

#### Data availability statement

The data that supports the findings of this study are available from the corresponding author upon reasonable request.

## Results

We tested 28 healthy controls and 33 OCD patients with a probabilistic reasoning task (Figure 1A). The task, adapted from a previous study (35), required the subjects to make two-alternative choices based on a sequence of stimuli. In each trial, the computer first randomly decided on a correct answer. The subjects then watched a sequence of visual stimuli displayed on a computer screen. The stimuli were arrows that were sampled randomly with replacement from a pool of ten arrows, the distribution of which depended on the correct answer (Figure 1B). Thereby, each arrow *A*_*i*_ was associated with two likelihoods (P[*A*_*i*_|*left*] and P[*A*_*i*_|*right*]), describing how likely one may observe the arrow given the correct answer being the left or the right. The subjects reported their choice by pressing the left or the right button whenever they felt ready. The sequence was cut off at the 100*th* arrow, after which the trial would be aborted if the subjects did not make a response.

We defined the arrows' weight as to the log ratio between the two likelihoods. The five right arrows had positive weights, indicating that they were evidence supporting the right choice. The five left arrows had negative weights and supported the left choice. Unlike the previous studies (35), in which the associations between the stimuli's visual features and their evidence weights were arbitrary, we used the arrow directions and contrasts to indicate their weights of evidence: larger contrasts were assigned with weights of larger absolute values, indicating stronger evidence. This design reduced the training process in which the subjects had to learn the weights assigned to the stimuli. Subjects only needed to combine evidence from the arrows in a trial by calculating the sum of the weights associated with them. The sum of the weights is the log odds between the right choice and the left choice being the correct choice (Eqs. 2 and 3).

**Figure 2:**
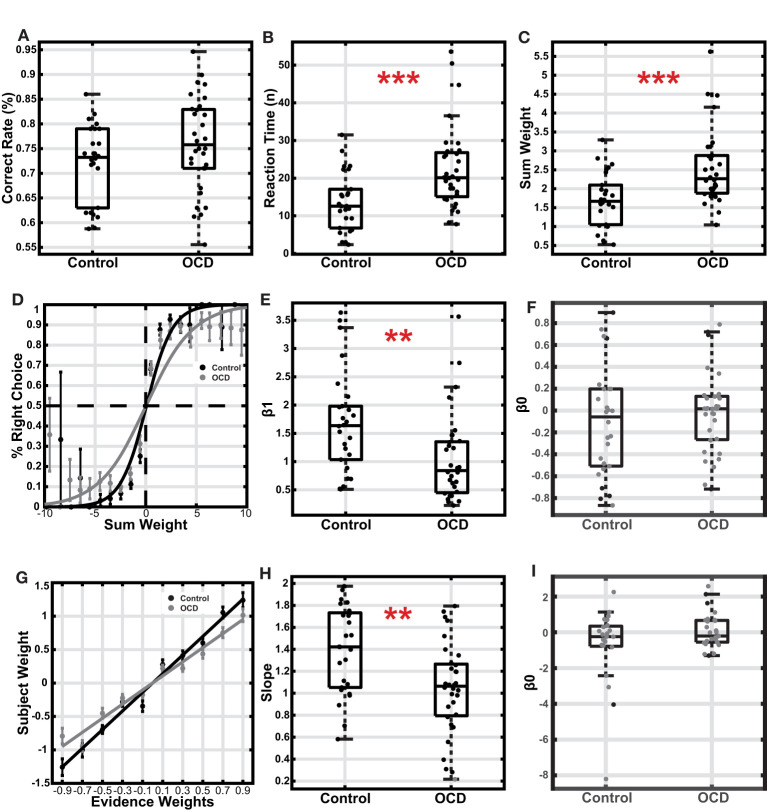
Comparison between the OCD and the control group. **(A)** Accuracy. The boxes indicate the second and third quartiles, and the whiskers indicate a 99.3% interval. **(B)** Reaction time is measured with the number of stimuli used for decisions. **(C)** The sum weight of the stimuli used for decisions. **(D)** The choice was plotted as a function of sum weight. The data points and the error bars indicated the mean and the S.E.M. across subjects. The curves were based on the logistic regression fitting to all trials of each group's subjects. **(E)** The logistic regression coefficient β1 of individual subjects. **(F)** The logistic regression coefficient β0 (intercept) of individual subjects. **(G)** The subjective weights plotted against the assigned weights for each type of arrow. The data points and the error bars indicated the mean and the S.E.M. across subjects. **(H)** The slopes of the fitted lines of the subjects' subjective weights. **(I)** The logistic regression coefficient β0 (intercept) of individual subjects. The gray dots indicated subjects with slopes not significantly different from zero, who were excluded from the analysis **p < 0.01, ***p < 0.001.

Most of the participants from both groups learned the task successfully. We excluded participants whose performance accuracy was outside of the 95% confidence interval. With this criterium, we included 26 healthy controls and 32 OCD patients in the following analysis. There were no significant differences between gender, age and the education of the two groups (Table 1; gender: x^2^(1, *N* = 58) = 0.0056, *p* = 0.94; chi-square test; age: t(56) = −0.98, *p* = 0.33, education: t(56) = 1.11, *p* = 0.27, *t*-test). There was no significant difference in either the mean or the variance between the accuracies of the OCD and the healthy control group (Figure 2A, Control (mean±SEM): 0.72 ± 0.01, OCD: 0.76 ± 0.02; t(56) = −1.72, *p* = 0.092, *t*-test; effect size = −0.447; 95%CI: [−0.970, 0.087], Hedges' g; Levene's test, *p* = 0.246).

**Table 1:** Characteristics of the participants.

	**Age**	**Gender (M/F)**	**Education**	**FOA**	**Dur**	**YB-O**	**YB-C**	**YB-T**	**HAM-D**	**HAM-A**
**Control**	26.27 (7.78)	16/10	16.12 (2.18)	n.a.	n.a.	0.77 (1.07)	0.50 (0.99)	1.27 (1.78)	1.35 (1.62)	0.81 (0.85)
**OCD**	28.25 (7.53)	20/12	15.38 (2.77)	21.31 (7.25)	7.28 (5.34)	11.00 (2.09)	9.91 (3.12)	20.91 (4.54)	11.41 (6.11)	7.91 (5.22)

Table above listed the mean and the standard deviations (in parentheses). FOA, first onset age; Dur, illness duration; YB-O, Y-BOCS-obsession; YB-C, Y-BOCS-compulsion; YB-T, Y-BOCS-total; HAM-A, Hamilton Anxiety Rating Scale; HAM-D, Hamilton Depression Rating Scale.

Next, we examined how the subjects accumulated evidence for decision-making. The evidence was furnished with a stream of stimuli in each trial. However, because the subjects made decisions while a continuous stream of stimuli was presented, the last few stimuli before the button pressing likely did not contribute to the decision-making due to the extra processing required by the brain after a decision was made. We used logistic regression to estimate the number of stimuli that did not contribute to the decision-making (see methods and Eq. 1). On average, the last 2.27 ± 0.32 stimuli for the control group and 2.41 ± 0.32 stimuli for the OCD group did not affect decision making and, therefore, were removed in the following analyses (Supplementary Figure 1).

After removing the stimuli that did not contribute to decision making, we found that the OCD patients looked at significantly more stimuli than the healthy controls before they committed to their decisions (Figure 2B, OCD: 22.54 ± 1.96, Control: 13.61 ± 1.52; t(56) = −3.47, *p* < 1e^−3^, *t*-test; effect size = −0.905; 95% CI: [−1.441 −0.344], Hedges' g; Levene's test, *p* = 0.221; error trials excluded). The total amount of evidence at the time of the decision, measured by the sum of stimulus weights of the stimulus sequence, was also relatively higher in the OCD group (Figure 2C, OCD: 2.55 ± 0.18, Control: 1.70 ± 0.14; t(56) = −3.63, *p* < 1e^−3^, *t*-test; effect size = −0.945; 95% CI: [−1.483, −0.381], Hedges' g; Levene's test, *p* = 0.26; error trials excluded). The fact that the OCD patients accumulated more evidence to achieve similar performance to the control group pointed to a lower decision-making efficiency in the OCD patients. The decision efficiency difference is also evident in the psychometric curves, which were steeper in the control group than in the OCD group (Figure 2D). A logistic regression model of the subject's choice revealed that the total evidence exerted less leverage on the OCD patients' choices than those of the healthy controls (Figure 2E, OCD: 1.04 ± 0.14, Control: 1.69 ± 0.18; z = 3.12, *p* = 0.0018, Wilcoxon rank-sum test), which leads to a less steep psychometric curve for the OCD patients. The OCD patients' performance was also more variable than the controls (Levene's test, *p* = 0.0446). Neither group showed a significant choice bias (Figure 2F, OCD(mean±standard deviation): −0.02 ± 0.36, t(31) = −0.30, *p* = 0.77; Control: −0.10 ± 0.50, t(25) = −1.05, *p* = 0.30; one sample *t*-test). Finally, we used another logistic regression model to study how each arrow type affected the subjects' choices. The regression coefficients termed the subjective weight of evidence (36), measure the weights that subjects assigned to each type of arrow based on their choice behavior. Although the ranking order of the subjective weights was consistent with the assigned weights in both groups (Figure 2G), the OCD patients' subjective weights were smaller than those of the healthy control. We further regressed the arrows' subjective weights against their true weights for each subject. The slope of the fitting line was significantly different from 0 in all but one subject from the OCD group (*p* < 0.05). The average slope of the OCD patients was smaller than that of the controls, even after excluding an OCD subject with a non-significant slope (Figure 2H; OCD: 1.07 ± 0.07, control: 1.39 ± 0.08; *p* = 0.0028, *t*-test; Levene's test, *p* = 0.280). Therefore, each piece of evidence contributed less to the choices in the OCD patients than in the healthy controls, suggesting the patients were less sensitive to the evidence. Again, the subjects in neither group exhibited a choice bias (Figure 2I; OCD(mean±standard deviation): 0.09 ± 0.97, t(26) = 0.46, *p* = 0.65, one sample *t*-test; Control: −0.68±2.08, t(23) = −1.60, *p* = 0.12, one sample *t*-test).

**Figure 3:**
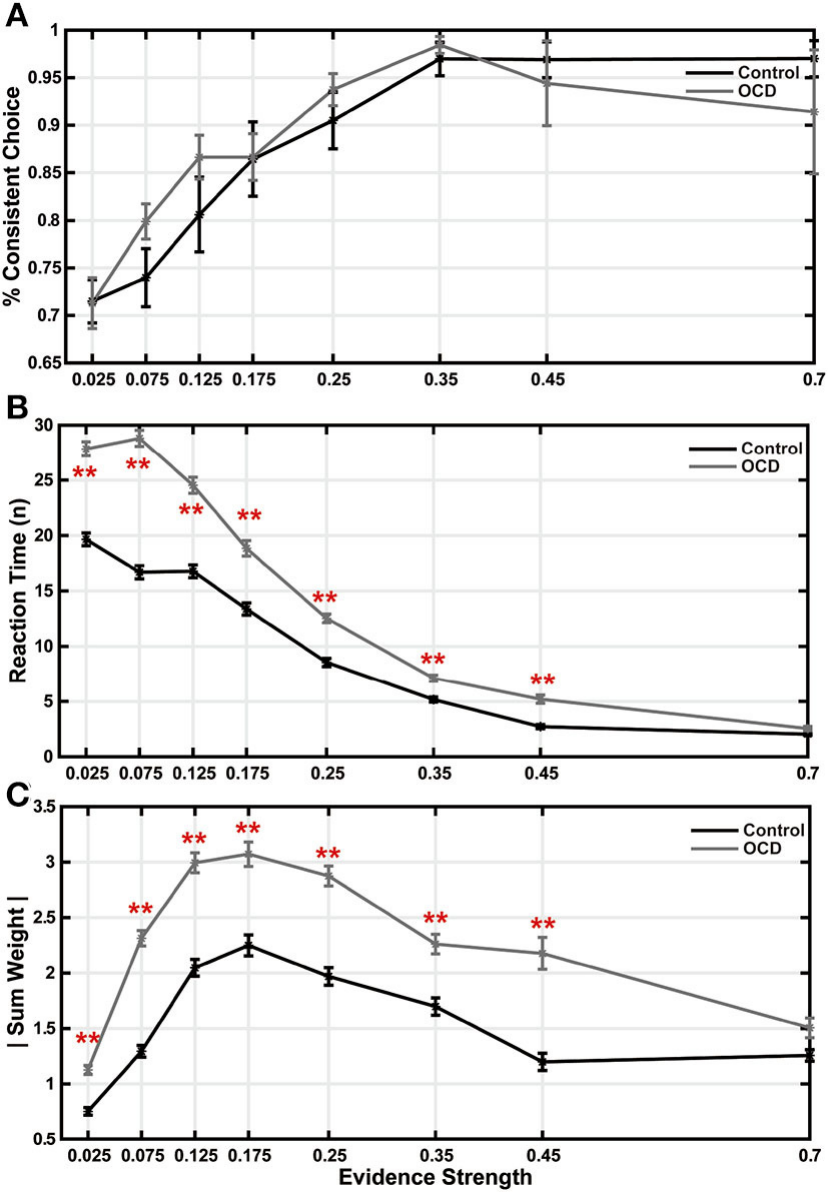
Trial difficulty affected decision making. **(A)** Accuracy is plotted as a function of evidence strength. There were no significant differences between the two groups. **(B)** Reaction time. **(C)** Sum weight. All the data points and the error bars indicate the mean and the S.E.M. across subjects (**p < 0.01, two-sample *t*-test, after multiple comparison correction).

The less evidence sensitivity in OCD patients leads us to suspect that their decision-making may be more impacted during difficult trials, which may be based on the accumulation of many pieces of weak evidence. Therefore, we studied how trial difficulty may affect evidence accumulation in OCD patients. The trial difficulty can be quantified by how fast the evidence grows in a trial, which we term evidence strength. We regressed the accumulated evidence weight against the stimulus index in each trial, excluding the non-contributing arrows, and defined the evidence strength as the slope of the regression line (Eq. 6). We grouped the trials by their evidence strengths. At each level of evidence strength, the choice accuracy did not show any significant difference between the two groups (Figure 3A). During the easiest trials, the OCD patients and the controls also took a similar amount of time to make their decisions (Figure 3B, last bin). However, the OCD patients spent significantly more time when the evidence strength was weak (Figure 3B). The difference became gradually larger as the evidence became weaker and the trials became harder except in the most difficult trials. When we looked at the amount of accumulated evidence at the time of the decision, both groups showed a non-monotonic pattern with the evidence strength increased. Subjects from both groups accumulated increasingly more evidence as the task difficulty elevated from easy to moderately difficult, but they collected less evidence when the difficulty became overwhelming (Figure 3C). During the easiest trials (the last bin in Figure 3C), the OCD and the control group accumulated a comparable amount of evidence. The trial numbers and participant numbers included in each were displayed in (Supplementary Table 1).

In addition, we observed no repetitive choice pattern from the OCD subjects. When we regressed the subjects' choice against the previous trial's choice, reward outcome, and their interactions (Eq. 7), we did not find any historical influences in either group at the group level (Supplementary Figure 2).

**Figure 4:**
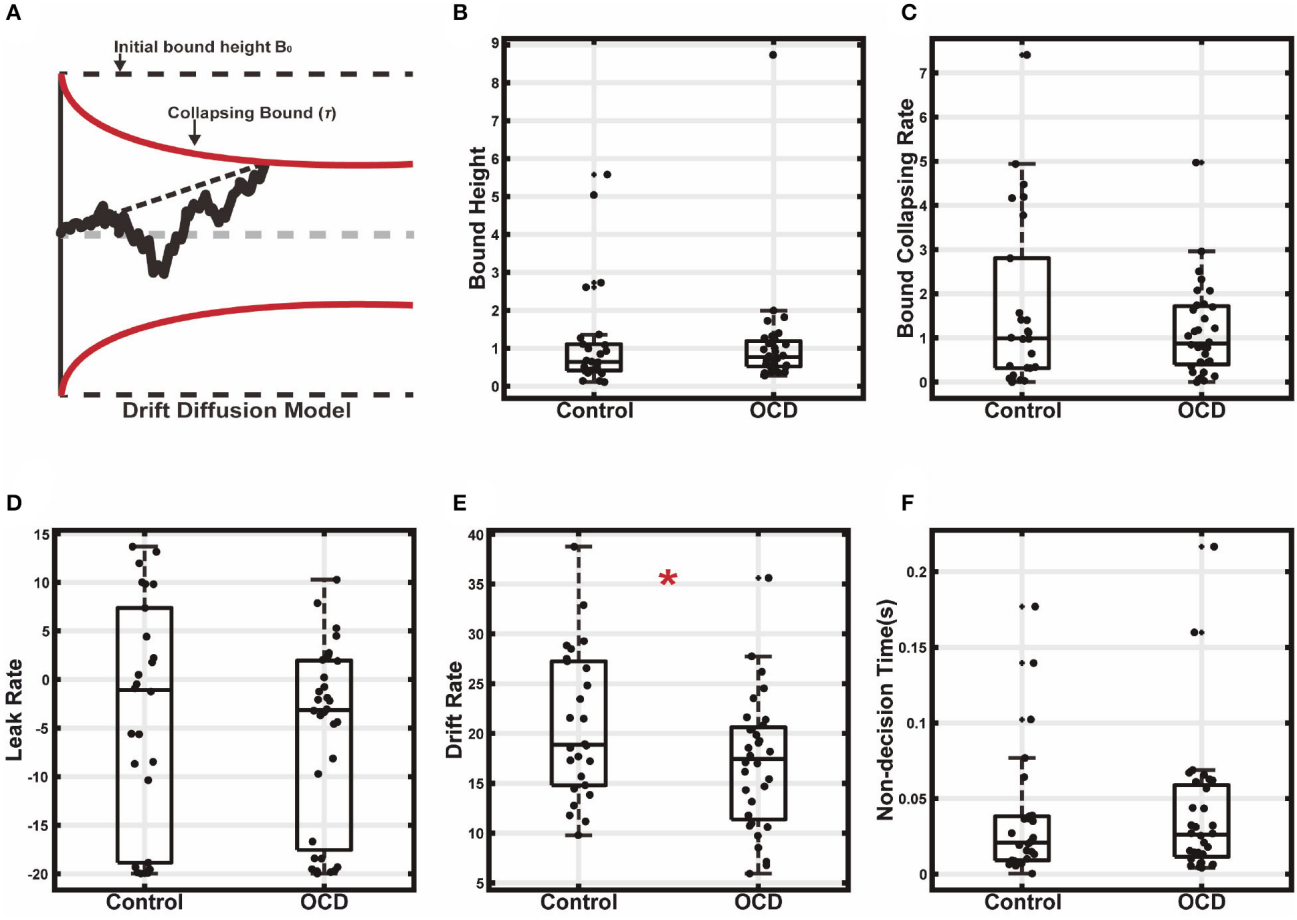
Drift diffusion model fitting. **(A)** The decision making was modeled as a leaky drifting process toward symmetric collapsing bounds. **(B)** Initial bound height. **(C)** Bound collapsing rate. **(D)** Leaky rate. **(E)** Drift rate. **(F)** Non-decision time (*p < 0.05).

These results indicated that the OCD patients had longer reaction times. It could be because they required more evidence for making decisions, or it could also be each stimulus provided a smaller amount of evidence, or both. The former means a higher decision threshold, and the latter leads to a slower evidence accumulation rate. To gain further insights into a mechanistic explanation of the OCD patients' decision-making, we used the drift-diffusion model (DDM) to infer the subjects' evidence accumulation process during the decision-making (44). The DDM is a model for explaining both the choice and reaction time behavior in two-alternative choice tasks. In the DDM, the decision-making is modeled as a particle drifting between two bounds. The drifting rate depends on the strength of the evidence, and the decisions are made when the particle hits one of the two bounds. We modeled the subjects' decision-making with a DDM in which the evidence accumulation process is leaky and the bounds collapse over time (Figure 4A). To simplify the model and speed up the fitting, the drifting rate in each trial is set to be fixed, which is proportional to the evidence strength, with the evidence sensitivity being the proportionality constant. The bounds collapse exponentially with a time constant τ. The details of the fitting procedures were described previously (45).

The model fitting results supported the second hypothesis that the longer reaction time of the OCD group was due to a less efficient evidence accumulation process. We found that the decision bound was similar between the two groups (Figure 4B, OCD (mean±SEM): 1.11 ± 0.26, control: 1.14 ± 0.27; t(56) = 0.09, *p* = 0.931, two-sample *t*-test). The bound collapsing rate τ was also comparable (Figure 4C, OCD: 1.16 ± 0.19, control: 1.68 ± 0.38; z = 0.11, *p* = 0.91, Wilcoxon rank-sum test). What was significantly different between the two groups was the evidence sensitivity, which was larger in the control (Figure 4E, OCD: 17.03 ± 1.17, control: 20.91 ± 1.45; t(56) = 2.10, *p* = 0.04, two-sample *t*-test). Therefore, the evidence was accumulated more quickly and reached the decision bound faster in the control group, leading to a shorter reaction time. The accumulation leak rate showed no difference between the OCD patients and the control (Figure 4D, OCD: −5.63±1.67, control: −3.61 ± 2.34; t(56) = 0.72, *p* = 0.475, two-sample *t*-test). We also did not find a difference in the non-decision time (Figure 4F, OCD: 0.040 ± 0.008, control: 0.040 ± 0.009; t(56) = −0.17, *p* = 0.86, two-sample *t*-test), which accounted for the time for extra sensory and motor processing for decision making, suggesting the OCD patients and the controls were similar in sensory processing and action execution speed.

We further used DDM with constant bound to fit the subjects' behavior (Supplementary Figure 3). As above in the model with collapsing bounds, the evidence sensitivity again showed a significant group difference (OCD (mean±SEM): 15.26 ± 0.98, control: 18.19 ± 1.04; t(56) = 2.03, *p* = 0.047, two-sample *t*-test). In addition, the leak rate was also found to be slightly but significantly smaller in the OCD group than in the control (OCD: 3.50 ± 0.84, control: 4.70 ± 1.96; z = 2.31, *p* = 0.021, Wilcoxon rank sum test). Bound height (OCD: 1.34 ± 0.13, control: 1.20 ± 0.16; t(56) = −0.69, *p* = 0.49, two-sample *t*-test) and non-decision time (OCD: 0.019 ± 0.006, control: 0.025 ± 0.007; t(56) = 0.63, *p* = 0.53, two-sample *t*-test) were comparable between the two groups. The DDM with collapsing bound was not significantly better than the DDM with constant bound in either the control or the OCD group (Supplementary Figure 4). (ΔAIC_control_ = 1.70, t(50) = −0.05, *p* = 0.96; ΔAIC_OCD_ = 3.62, t(62) = −0.15, p = 0.88; ΔBIC_control_ = −0.89, t(50) = 0.03, *p* = 0.98; ΔBIC_OCD_ = 1.04, t(62) = −0.04, *p* = 0.97).

Finally, we studied how the individual variations of the OCD patients' performance may correlate with their disease symptoms' severity and characteristics (Supplementary Table 2). Each patient completed the tests of the Yale-Brown Obsessive-Compulsive Scale (Y-BOCS) (40), Hamilton Anxiety Rating Scale (HAM-A) (41), and Hamilton Depression Rating Scale (HAM-D) (42). We observed several trends that may indicate a correlation between the patients' decision-making and their symptoms. In particular, the patients with higher compulsive symptoms, measured with Y-BOCS-C had less accuracy (Pearson's r = −0.31, *p* < 0.1) and shorter reaction time (Pearson's r = −0.31, *p* < 0.1). Similarly, the patients' HAM-A scores were also negatively correlated with both the reaction time (Pearson's r = −0.32, *p* < 0.1) and the sum weight (Pearson's r = −0.32, *p* < 0.1), as well as a positive correlation between the HAM-A score and the decision bound's collapsing rate (tau) (Pearson's r = 0.4, *p* < 0.05). The results indicate that higher anxiety levels may lead to hasty decisions in OCD patients. However, none of these correlations survive after multiple-comparison corrections. One possible reason is that the OCD cohort had a small range of symptom severity level, which is not sufficient to reveal the correlation between the symptom severity and the decision-making deficits. In addition, the patients' impaired sensory-evidence transformation may affect their symptoms *via* a complicated mechanism that cannot be detected *via* a simple correlation analyses. Further investigations are necessary to understand the relationship between the OCD symptoms and the altered cognitive functions.

## Discussion

We studied and compared the evidence accumulation during decision-making in OCD patients and controls. Consistent with previous studies, the OCD patients had longer reaction times than the healthy controls but achieved similar overall accuracy by collecting more evidence (24). However, the difference in the reaction time was not evident when the trials were easy. We demonstrated that the longer reaction times were due to OCD patients' less efficiency in using the evidence for decision making.

The behavior paradigm used in our study has several distinct features. First, unlike the random dots motion task, which has been extensively used in both primate and human studies (24, 37, 39), the stimuli were highly discriminable in the current experiment. Therefore, our results are not confounded by the perceptual limits. In addition, unlike a monkey study with a similar concept (35), the stimulus-evidence association was not entirely arbitrary. Instead, the arrows of different directions and contrast levels indicated the evidence strength and the correct target. Thereby, we lowered the memory demand and speeded up the learning of the task. This is particularly helpful in the patient study due to a stricter limit of trial numbers. Finally, the amount of evidence can be directly calculated from the weights of the stimuli that appear in each trial, allowing us to explore the evidence accumulation process during decision making.

In particular, the accumulated evidence at the time of the decision, measured with the sum of weights, is not the same as the decision bound determined by the DDM. While the decision bound reflects an internal threshold used for decision making, the evidence weight, and its sum is an objective measure of the amount of evidence collected. The evidence sensitivity determines how external evidence is transformed and used in the brain for decision making. We used a DDM to show that the evidence sensitivity differed between the OCD patient group and the healthy control group, suggesting that the same amount of evidence contributed less to decision-making in the OCD patients.

This deficiency in OCD patients does not necessarily mean that they were worse at perceiving visual stimuli. There is no strong evidence suggesting that OCD patients' sensory perception is compromised. Instead, we believe that it reflects a deficit in the transformation from the sensory stimuli into the evidence in OCD patients. The same stimulus appears to be less informative to the patients than to the healthy controls, which may lead to their apparent indecisiveness.

By calculating the sum weight at the decision time, we found that the total accumulated evidence first increased with the trial difficulty but collapsed at extreme difficulty levels. This trend was not seen in the reaction time, which increased monotonically with the trial difficulty. At the easiest difficulty level, the OCD patients accumulated a similar amount of evidence to that of the healthy controls. Yet, the increase of the accumulated evidence with the trial difficulty was significantly higher among the OCD patients than among the healthy controls, reflecting that the OCD patients' decision-making is more impaired under challenging situations.

Greater trial difficulties lead to more uncertain and less confident decisions. The estimation of confidence may allow the brain to adjust decision strategies dynamically during decision-making. It has also been proposed that confidence may be estimated in a metacognition process based on the same evidence accumulation process during the decision making (46, 47). Also, a prefrontal network that includes the orbitofrontal cortex and the anterior cingulate cortex was implicated (48–50). In particular, a recent study using a similar task showed that the orbitofrontal cortex encodes evidence associated with each stimulus, while the dorsolateral prefrontal cortex transforms evidence into action value and accumulates evidence for making motor responses (28). Our results suggest a deficit in the orbitofrontal cortex among OCD patients, which has been demonstrated previously (51–53).

Therefore, we suspect that an abnormal interplay between the prefrontal metacognition circuitry and the decision-making circuitry, which includes the posterior parietal cortex and basal ganglia, underlies the observed decision-making behavior in OCD patients. Consistent with this idea, a recent study showed that capsulotomy, which affected cortical-basal-ganglia projections, could rectify some of the decision-making problems in OCD patients (54).

We only observed weak correlations between the OCD patients' decision-making and the severity of their symptoms, which were measured with Y-BOCS, HAM-A, and HAM-D scores. These correlations did not point to a consistent behavior pattern. For example, while higher compulsivity seems to lead to shorter reaction times and worse accuracies, patients with higher anxiety levels, although also exhibiting shorter reaction times, were more sensitive to the evidence, and their accuracies were less affected. The link between the patients' symptom and their decision-making behavior may be complex, and multiple factors may be in play. Further studies are necessary to confirm these findings.

In conclusion, we used a novel probabilistic reasoning task to explore the decision-making behavior in OCD patients. The results revealed that the deficiency in OCD patients' decision-making is due to an inefficient accumulation of evidence. With decision bounds similar to those of the controls, the OCD patients, however, could compensate for this deficiency with longer reaction times to reach comparable accuracies.

## Data availability statement

The raw data supporting the conclusions of this article will be made available by the authors, without undue reservation.

## Ethics statement

The studies involving human participants were reviewed and approved by the Ethics Committee of Shanghai Mental Health Center (Shanghai, China). The patients/participants provided their written informed consent to participate in this study.

## Author contributions

TY, ZW, QF, and YC: conceptualization. YL: data acquisition. TY, YC, QF, and YL: data analysis. TY and YC: drafting. TY, QF, and YC: final approval. TY and QF: funding and supervision. All authors contributed to the article and approved the submitted version.

## Funding

This work was supported by the National Key R&D Program of China (No. 2019YFA0709504), National Science and Technology Innovation 2030 Major Program (No. 2021ZD0203701), Shanghai Municipal Science and Technology Major Project (No. 2018SHZDZX05), the Strategic Priority Research Program of Chinese Academy of Science (No. XDB32070100 to TY), the National Natural Science Foundation of China (Nos. 81771460 and 81671340), Scientific Project from Shanghai Municipal Health Bureau (No. 201740086), Clinical Medicine Project of Shanghai Science and Technology Committee Project (No. 18411952000), Key Laboratory of Psychotic Disorders (No. 13dz2260500), and Innovative Research Team of High-Level Local Universities in Shanghai to QF.

## Conflict of interest

The authors declare that the research was conducted in the absence of any commercial or financial relationships that could be construed as a potential conflict of interest.

## Publisher's note

All claims expressed in this article are solely those of the authors and do not necessarily represent those of their affiliated organizations, or those of the publisher, the editors and the reviewers. Any product that may be evaluated in this article, or claim that may be made by its manufacturer, is not guaranteed or endorsed by the publisher.
