# Supplementary material for: Accumulation of evidence during decision making in OCD patients
Source: Front Psychiatry. 2022 Sep 23;13:980905. doi: 10.3389/fpsyt.2022.980905 (PMC9539281; doi:10.3389/fpsyt.2022.980905)
Supplement: Supplementary file 1 [file Data_Sheet_1.pdf]

Supplementary Figures

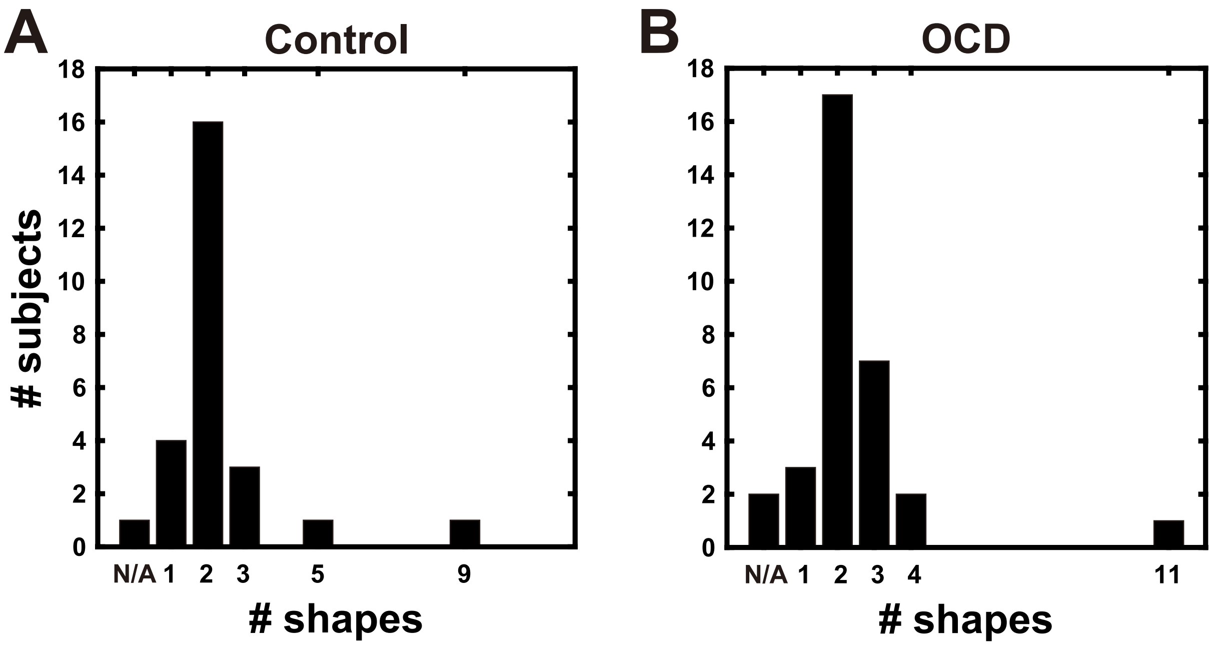

Sup Figure 1. Number of non-decision stimuli. (**A**) Control group. (**B**) OCD group. The histograms of the subjects' non-decision stimulus number. N/A indicates the subjects whose non-decision stimulus number cannot be determined with the current method.

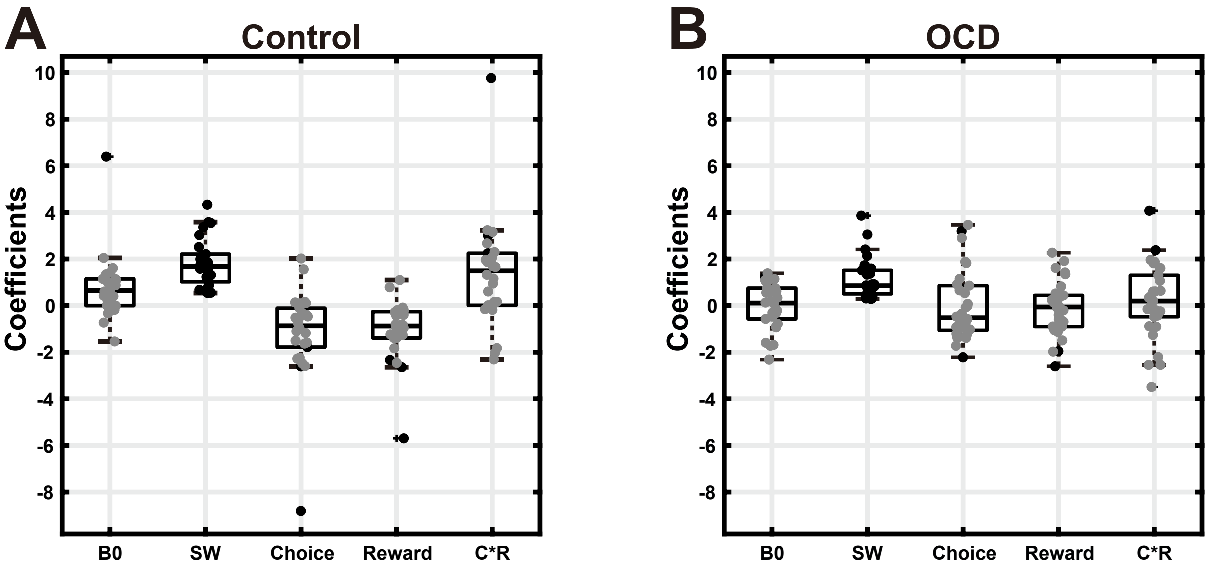

Sup Figure 2. Trial history effects. (A) Control group. **(B)** OCD group. Gray dots indicate coefficients not significantly different from 0 ($p>0.05$), black dots indicate significance ($p<0.05$). Only the summed weight (SW) showed significant effects on the choice consistently among the subjects. Two subjects in the OCD group were not fit well to the regression model and were excluded in the plot.

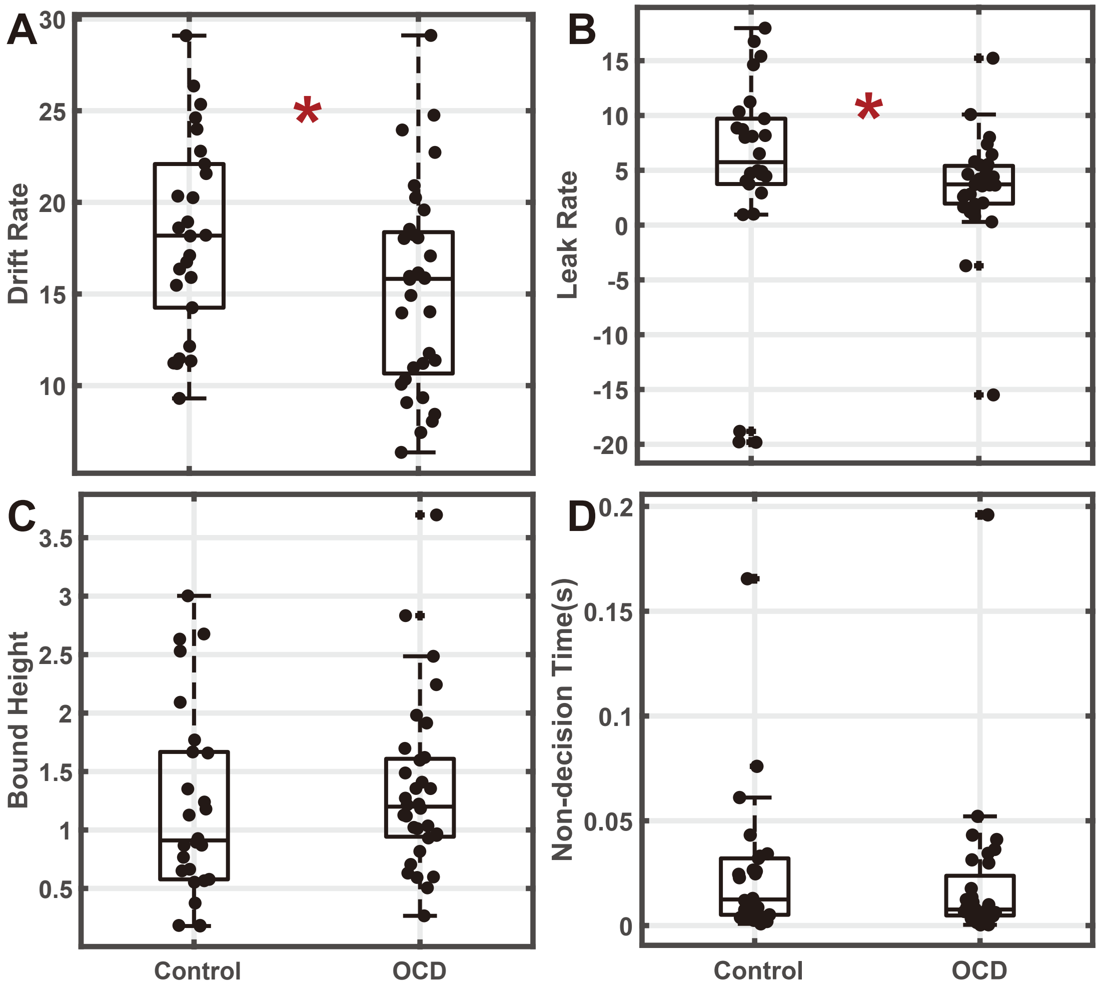

Sup Figure 3. Drift diffusion model fitting with a constant bound. The decision making was modeled as a leaky drifting process toward symmetric constant bounds. (a) Drift rate. (b) Leaky rate. (c) Bound height. (d) Non-decision time. (* $p<0.05$)

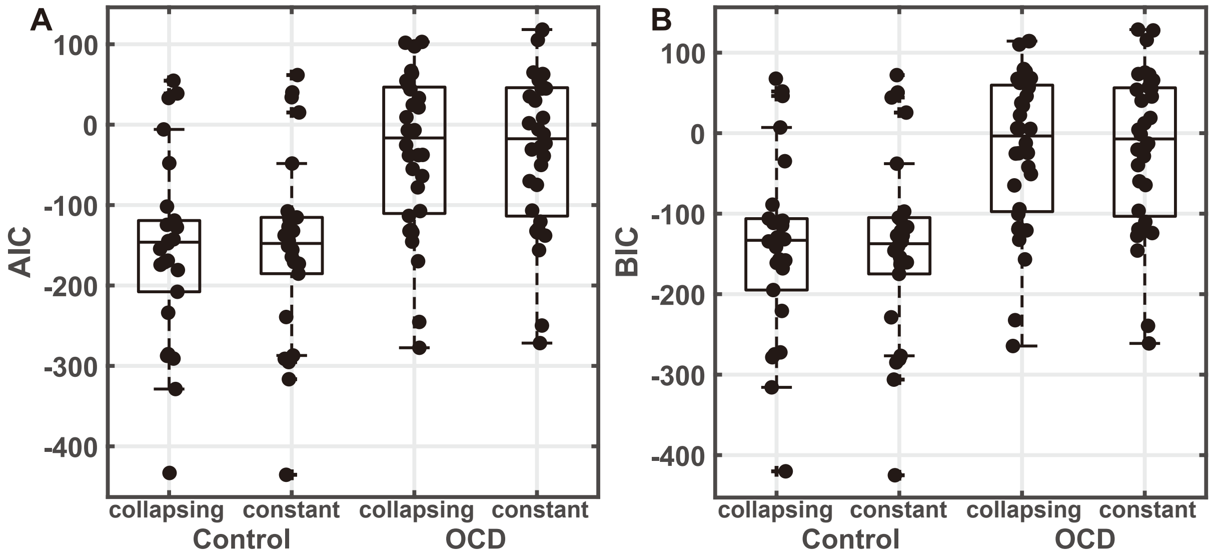

Sup Figure 4. Model comparison, AIC and BIC of the DDM with a collapsing bound and a constant bound. Drift diffusion model fitting. (a) AIC of the DDM with a collapsing bound and with a constant bound. (b) BIC score of the DDM with a collapsing bound and with a constant bound.

Supplementary Tables

Evidence Strength		0-0.05	0.05-0.1	0.1-0.15	0.15-0.2	0.2-0.3	0.3-0.4	0.4-0.5	0.5-max
#trials	Control	528	513	468	363	402	202	90	34
	OCD	703	716	634	430	440	145	53	13
#subjects	Control	26	26	26	26	26	25	21	12
	OCD	32	32	32	32	32	29	19	8

Supplementary Table 1. Evidence strength binning. In the first row are the ranges of each bin. The second and third rows are the number of trials that go into each bin for the control and the OCD group, respectively. The last two rows show the number of subjects that contribute to each bin.

Correlations - OCD group										
	**CR**	**RT**	**SW**	**PC**	**SV**	**ES**	**leak**	**B**	**tau**	**nd**
**Y-BOCS-O**	-0.07	0.12	0.12	-0.17	0.16	-0.15	-0.14	-0.2	0.13	-0.2
**Y-BOCS-C**	-0.31*	-0.31*	-0.27	-0.16	-0.06	-0.23	-0.01	-0.18	0.09	-0.2
**Y-BOCS-T**	-0.25	-0.16	-0.13	-0.19	0.04	-0.22	-0.07	-0.22	0.13	-0.23
**HAMD**	-0.15	-0.26	-0.25	-0.04	0.1	-0.02	0.03	0.04	0.2	-0.2
**HAMA**	-0.18	-0.32*	-0.32*	0.08	0.3*	0.06	0	-0.12	0.4**	-0.21

Supplementary Table 2. Symptom correlations. The correlation between the OCD patients' task performance and their symptom severity measured by Y- BOCS-C and HAM-A. Abbreviations: Y-BOCS-O: Y-BOCS-Obsession; Y- BOCS-C: Y-BOCS-Compulsion; Y-BOCS-T: Y-BOCS Total; HAMD: Hamilton Depression Rating Scale; HAMA: Hamilton Anxiety Rating Scale; CR: correct rate; RT: reaction time; SW: sum weight; PC: β1 of the logistic fitting of the psychometric curve; SV: the slope of the fitted line of the subjective values against the assigned weights. ES: evidence sensitivity; leak: leak rate; B: initial bound height; tau: bound collapsing rate; nd: non-decision time. * Indicates $p<0.1$, ** indicates $p<0.05$. The p-values were not adjusted for multiple comparison.
